# Effects of different potassium-lowering regimens on acute hyperkalemia in hemodialysis patients: a real-world, retrospective study

**DOI:** 10.1186/s12967-022-03530-4

**Published:** 2022-07-25

**Authors:** Lan Yao, Xiaoyang Xing, Yubao Li, Fangxing Zhang, Ping Li, Xianhui Liang, Pei Wang

**Affiliations:** 1 Blood Purification Center, Department of Nephrology, The First Affiliated Hospital of Zhengzhou University, 1 East Jianshe Road, Zhengzhou, 450052 Henan China; 2 Research Institute of Nephrology, Zhengzhou University, Zhengzhou, 450052 China; 3 Blood Purification Center, The First Affiliated Hospital, and College of Clinical Medicine of Henan University of Science and Technology, Luoyang, 471003 China

**Keywords:** Sodium zirconium cyclosilicate, Hyperkalemia, Hemodialysis, Chronic kidney disease, Potassium-lowering regimens

## Abstract

**Background:**

Hyperkalemia is a common and potentially life-threatening electrolyte disorder in maintenance hemodialysis (MHD) patients. This study aimed to evaluate the efficacy and safety of potassium-lowering regimens during treatment of acute hyperkalemia in MHD patients.

**Methods:**

This retrospective real-world study (RWS) was conducted among 139 MHD patients. They were given different potassium-lowering regimens, viz. the insulin and glucose (IG) intravenous administration group (IG, 46 patients), the sodium polystyrene sulfonate group (SPS, 33 patients), the sodium zirconium cyclosilicate group (SZC, 38 patients), the IG + SZC group (22 patients). The primary efficacy end point was the rate of serum potassium decline at 2 h. The rates of adverse events were also compared.

**Results:**

At 2 h, the mean ± SE change of serum potassium level was − 0.71 ± 0.32 mmol per liter (mmol/L) in IG group, − 0.43 ± 0.38 mmol/L in SPS group, − 0.64 ± 0.36 mmol/L in SZC group, − 1.43 ± 0.38 mmol/L in IG + SZC group (*P* < 0.01). The serum potassium level in IG + SZC group decreased more than that in the other three groups (*P* < 0.01), while the serum potassium level in SPS group decreased less than that in the other three groups (*P* < 0.05). There was no significant difference on the decrease of the serum potassium level between IG group and the SZC group (*P* = 0.374). The IG group and the IG + SZC group had higher rates of symptomatic hypoglycemia. The SPS group had significant decreases of serum calcium and serum magnesium after treatment.

**Conclusions:**

Among MHD patients with acute hyperkalemia, SZC had similar potassium-lowering efficacy with IG intravenous administration at 2 h and superior on convenience and side-effects.

**Supplementary Information:**

The online version contains supplementary material available at 10.1186/s12967-022-03530-4.

## Introduction

Hyperkalemia is a common and potentially life-threatening electrolyte disorder in patients with chronic kidney disease (CKD), especially in those receiving maintenance hemodialysis (MHD) [1–3]. It is defined as serum potassium (K+) level > 5.5 mmol/L, regardless of whether patients receive an adequate treatment with hemodialysis (HD) [4, 5]. There occurs a decrease in the transmembrane K+ gradient in hyperkalemia which results in reduced ventricular conduction, cell membrane depolarization, and a decrease in the duration of myocardial action potential [6]. Additional risk factors for hyperkalemia in CKD patients were associated with heart failure, hypertension, diabetes, metabolic acidosis, non-adherence to dietary restrictions, advanced renal impairment and use of renin–angiotensin–aldosterone system inhibitors [1, 2, 7].High serum potassium is usually corrected in a 3–5 h hemodialysis session, consequently the MHD patients regularly experience wide alterations in serum potassium levels, which at the extremes can reach pathologically high or low concentrations. Pre-dialysis hyper-and hypokalemia increases the risk of cardiovascular disease and sudden cardiac death, which is a serious complication endangering the life of MHD patients [7, 8]. Therefore, it is necessary to take emergency measures to reduce serum potassium level to a safe range for the management of acute hyperkalemia.

**Fig. 1:**
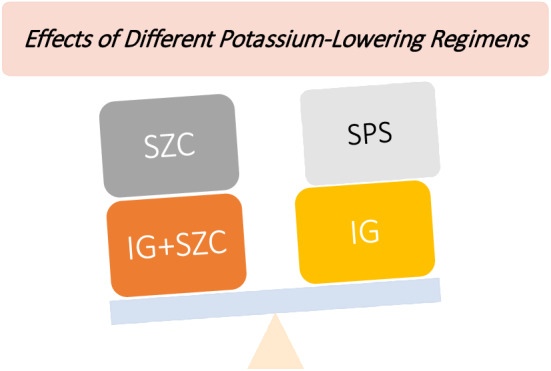
Effects of different potassium-lowering regimens on acute hyperkalemia in hemodialysis patients

There are a variety of potassium reduction programs in the acute phase of hyperkalemia, which however are currently lacking controlled trials to guide treatment decisions. The main agents used to correct the serum potassium levels and correcting hyperkalemia include the administration of intravenous insulin and glucose, cation resins such as sodium polystyrene sulfate (SPS), sodium zirconium cyclosilicate (SZC) and the combination of two or more treatment measures [5, 9–11]. The clinical studies on agents have reported variable results in reducing serum potassium levels in patients with acute hyperkalemia. However, there is no controlled study on the efficacy and safety of these regimens in the correcting of acute hyperkalemia. Therefore, the objective of this retrospective real-world study (RWS) was to evaluate the efficacy and safety of various regimens for the treatment of acute hyperkalemia in MHD patients (Fig. 1).

## Materials and methods

### Study population

This was a retrospective, observational, single center, real-world study conducted from June 10, 2020 to February 3, 2021, in which adult MHD patients with hyperkalemia (venous serum potassium > 5.5 mmol/L) were admitted in the Department of Nephrology of the First Affiliated Hospital of Zhengzhou University. We conducted our study using de-identified patient data; This study adhered to the Declaration of Helsinki and informed consent was not required and used anonymized patient data, The approval number of the ethics committee is 2021-KY-0664-001.

### Inclusion and exclusion criteria

**Fig. 2:**
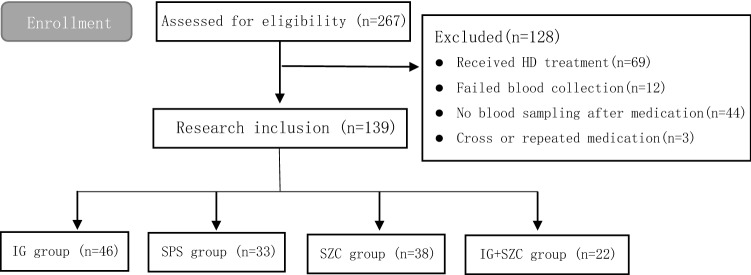
Patient flow diagram of study cohort and excluded patients

The following inclusion criteria were used to identify our patient population: men or women aged ≥ 18 years on MHD for ≥ 3 months and with serum potassium ≥ 5.5 mmol/L. Patients with erroneous collection method and those using other potassium-lowering drugs within 24 h before enrollment, and with repeated administration were excluded from the study. The case screening process is shown in Fig. 2.

### Methods and measurements

Electronic medical records were used to obtain clinical data. The general information of the patients such as age, gender, urine volume, major complications, etc., were collected from the hospital registry. The HD conditions such as function of vascular access, frequency of dialysis session, interval from the last dialysis and duration of the last dialysis were also analyzed. All concomitant medications were recorded such as angiotensin converting enzyme inhibitor (ACEI), angiotensin II receptor antagonist (ARB) or mineralocorticoid receptor antagonist (MRA). The serum levels of electrolytes (potassium, sodium, magnesium, calcium), glucose, and electrocardiogram (ECG) before and 2 h after treatment were also examined.

Patients were given 10 IU insulin plus 60 g glucose by intravenous drip (IG group), 15 g sodium polystyrene sulfonate (SPS group) orally, 10 g sodium zirconium cyclosilicate (SZC group) orally or IG + SZC (IG + SZC group) according to the clinical condition and doctors’ judgment. The serum level of potassium, whether typical findings on ECG or signs of hypervolume or relatively lower serum glucose level existed, were the main concerns (Additional file 1: Fig. S1). Peripheral blood samples, which were drawn on the precise timing point of 2 h after administration of these drugs, were sent for serum electrolyte and glucose test. All enrolled patients were not allowed to take food or medications influencing serum potassium.

### Study outcomes

Data extraction was completed by unblinded study investigators. The primary efficacy end point was assessed in terms of the exponential rate of change in the mean serum potassium level, standard rate of serum potassium (intravenous serum potassium < 5.5 mmol/L) and control rate of severe hyperkalemia after 2 h of treatment (from severe hyperkalemia to normal, moderate or mild hyperkalemia).

The main safety endpoints included assessment of adverse events (AEs); changes in laboratory parameters, including assessment of hypoglycemia or low blood sugar and the change rate of serum sodium, calcium and magnesium 2 h after treatment. Severe hyperkalemia refers to venous serum potassium ≥ 6.5 mmol/L with typical ECG changes; moderate hyperkalemia refers to venous serum potassium ≥ 6.0 mmol/L and < 6.5 mmol/l without typical ECG changes [12]. Whereas, mild hyperkalemia refers to venous serum potassium 5.0 < 5.5 mmol/L [13]. Typical ECG changes refer to a symmetrical high sharp T wave, prolonged PR interval, decreased P wave amplitude and widened QRS wave on ECG [14]. Hypocalcemia was defined as corrected serum calcium < 2.1 mmol/L. Hypermagnesemia refers to the serum magnesium level > 1.25 mmol/L. Poor vascular access function refers to the failure of effective HD treatment due to internal fistula thrombosis, severe fistula stenosis and poor dialysis catheter function. Symptomatic hypoglycemia refers to palpitation, pale face, sweating, hunger and other symptoms during the treatment.

### Statistical analysis

Statistical analysis was performed using SPSS 24.0 software. Normally distributed data were expressed as mean and standard deviation while non normally distributed data were expressed as median and interquartile range (IQR). before and after treatment, paired Wilcoxon rank sum test (non-normal distribution) or paired t test were used for comparison, and rank sum test (non-normal distribution) or one-way analysis of variance (one-way ANOVA) were used for comparison among multiple groups. Kruskal–Wallis rank sum test (non-normal distribution) or LSD test were used for pairwise comparison. The description of qualitative data was expressed in percentage, and the difference of qualitative data was compared by *X*^2^ test or Fisher exact probability method, *P* < 0.05 was considered as statistically significant.

## Results

### Characteristics of study participants

139 patients were treated by potassium lowering drugs, among which 90.7% were unable to receive HD treatment due to vascular access dysfunction. Among the 139 patients, 81 were males and 58 were females, with an average age of 53.63 ± 14.62 years, 129 patients had access dysfunction, 47 patients had diabetes mellitus and 65 patients had heart failure. There were no significant differences in age, gender, urine output, dialysis frequency, complications and drug combination among the groups (*P* > 0.05 for all the groups). There were significant differences in the time from the last HD between IG group and SPS group (*P* = 0.016) (Table 1).

**Table 1:** Baseline characteristics of MHD patients with acute hyperkalemia according to the study group

	IG group	SPS group	SZC group	IG + SZC group	*P*
Number of people (cases)	46	33	38	22	
Age (years, x̄ ± s)	53.09 ± 14.22	52.52 ± 12.52	55.37 ± 17.38	53.41 ± 13.83	0.853
Gender (cases/%)					0.093
Male	29 (63.0)	13 (39.4)	25 (65.8)	14 (63.6)	
Female	17 (37.0)	20 (60.6)	13 (34.2)	8 (36.4)	
Combined with poor access function (cases/%)	43 (93.5)	27 (81.8)	34 (89.5)	22 (100)	0.452
Serum potassium (cases/%)					< 0.001^a^
5.5–5.9 mmol/L	30 (65.2)	17 (51.5)	19 (50.0)	2 (9.1)	
6.0–6.4 mmol/L	12 (26.1)	11 (33.3)	14 (36.8)	6 (27.3)	
≥ 6.5 mmol/L	4 (8.7)	5 (15.2)	5 (13.2)	14 (63.6)	
Changes of ECG hyperkalemia (cases/%)	21/46 (45.7)	12/26 (46.2)	16/30 (53.5)	15/20 (75.0)	0.148
Severe hyperkalemia	7/46 (15.2)	10/31 (32.3)	12/37 (32.4)	18/21 (85.7)	< 0.001^a^
Urine volume < 400 ml/d	39 (84.8)	27 (81.8)	29 (76.3)	17 (77.3)	0.766
Dialysis frequency (cases/%)					0.543
≥ 3 times/week	32 (69.6)	25 (75.8)	25 (65.8)	18 (81.8)	
< 3 times/week	14 (30.4)	8 (24.2)	13 (34.2)	4 (18.2)	
Time from last dialysis (cases/%)					0.016^b^
≤ 24 h	23 (50.0)	17 (51.5)	8 (21.1)	6 (27.3)	
24–72 h	13 (28.3)	8 (24.2)	14 (36.8)	8 (36.4)	
≥ 72 h	10 (21.7)	8 (24.2)	16 (42.1)	8 (36.4)	
Last dialysis < 4 h (cases/%)	7 (15.2)	4 (12.1)	5 (13.2)	3 (13.6)	0.789
Complications (cases/%)					
Hypocalcemia	6 (13.0)	10 (30.3)	12 (31.6)	2 (9.1)	0.052
Hypermagnesemia	12 (26.1)	12 (36.4)	14 (36.8)	10 (45.5)	0.432
Diabetes	18 (39.1)	13 (39.4)	12 (31.6)	4 (18.2)	0.317
Cardiac function classification					0.968
Grade I	9 (19.6)	8 (24.2)	8 (21.1)	4 (18.2)	
Grade II	5 (10.9)	4 (12.1)	3 (7.9)	2 (9.1)	
Grade III	7 (15.2)	6 (18.2)	6 (15.8)	2 (9.1)	
Grade IV	0 (0.0)	0 (0.0)	1 (2.6)	0 (0.0)	
Combined medication (cases/%)					0.958
ACEI	5 (10.9)	2 (6.1)	2 (5.3)	1 (4.5)	
ARB	7 (15.2)	6 (18.2)	6 (15.8)	4 (18.2)	
MRA	0 (0.0)	0 (0.0)	0 (0.0)	1 (0.0)	

^a^There was statistical differences between the SZC + IG group and the other three groups in the pairwise comparison, *P* < 0.01, the difference between the remaining groups was not statistically significant

^b^There was a statistical difference between the IG group and the SZC group in the pairwise comparison, *P* = 0.038

### Baseline serum potassium level

**Fig. 3:**
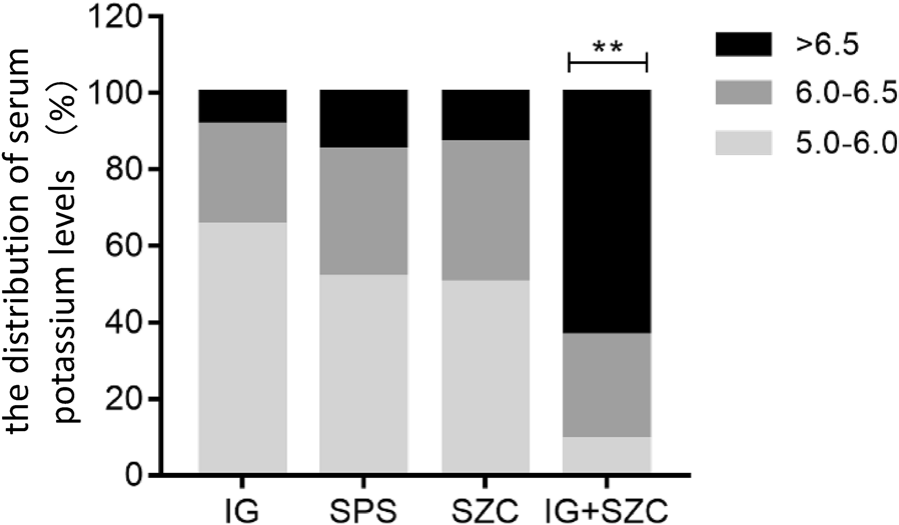
The distribution of serum potassium levels in each group, ** is the comparison of IG + SZC compared with the other three groups, *P* < 0.01

There was a significant difference in the baseline serum potassium level of each group before potassium lowering treatment (*H* = 27.32, *P* < 0.01). IG + SZC group had significantly higher serum potassium level than that of the other three groups (Fig. 3). Among the other three groups, the serum potassium level of SZC group was the highest, but there was no significant difference compared with the other two groups (*H* = 2.90, *P* = 0.235). In the IG + SZC group, 63.6% of patients had serum potassium ≥ 6.5 mmol/L and 85.7% of them had severe hyperkalemia, which was significantly higher than those in the other three groups (Table 1).

### Primary efficacy outcome: the rate of serum potassium decline

**Fig. 4:**
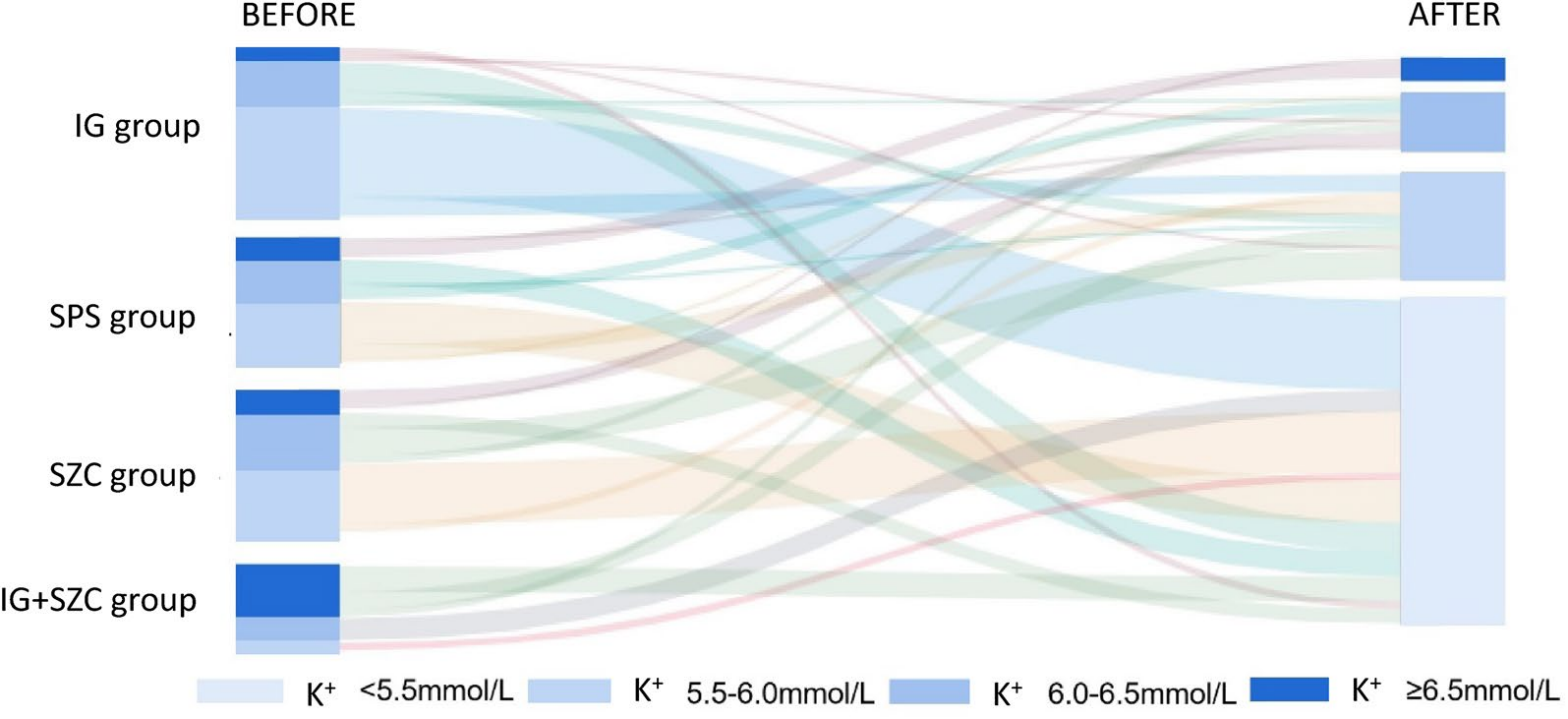
Sankey diagram of changes in serum potassium levels after treatment in each group

After treatment, there were statistical differences in serum potassium levels among groups (*H* = 10.28, *P* = 0.016) (Fig. 4 and Table 2). However, the differences between any two groups were not statistically significant except a significantly lower serum potassium level in the IG group than that in the SPS group (*P* = 0.017). Although the serum potassium level of SZC group was lower than that of SPS group after treatment, there was no statistical significance (*P* > 0.05) (Table 2).

**Table 2:** Changes of serum potassium(mmol/L) before and after treatment

	IG group	SPS group	SZC group	IG + SZC group	*P*
Serum potassium before treatment, M (Q1, Q3)	5.85 (5.64,6.09)	5.96 (5.70,6.24)	6.01 (5.74,6.27)	6.56 (6.37,7.08)	< 0.001^a^
Serum potassium after treatment M (Q1, Q3)	5.20^c^ (4.90,5.49)	5.38^c^ (5.27,6.07)	5.32^c^ (5.00,5.74)	5.30^c^ (4.65,5.53)	0.016^b^
Decrease of serum potassium	0.71 ± 0.32	0.43 ± 0.38	0.64 ± 0.36	1.43 ± 0.38	< 0.001^a^
Dispersion coefficient (CV) of serum potassium decline	45%	89.3%	56.8%	26.9%	
The rate of serum potassium decline (%)	11.89 ± 5.19	7.12 ± 6.32	10.58 ± 6.07	21.51 ± 5.74^a^	< 0.001
The standard-reaching rate of serum potassium (cases/%)	35 (76.1%)	19 (57.6%)	21 (55.3%)	15 (68.2%)	0.176
Control rate of severe hyperkalemia (cases/%)	7 (100%)	6 (60%)	11 (91.7%)	18 (100%)	0.135^d^

^a^There was no significant difference between IG and SZC groups in pairwise comparison, and the rest were statistically significant (*P* < 0.05)

^b^In pairwise comparison, there was no significant difference except IG group and SPS group (*P* < 0.05)

^c^Statistically significant difference compared with pretreatment (*P* < 0.05)

^d^*P* value of SPS group compared with SZC group

**Fig. 5:**
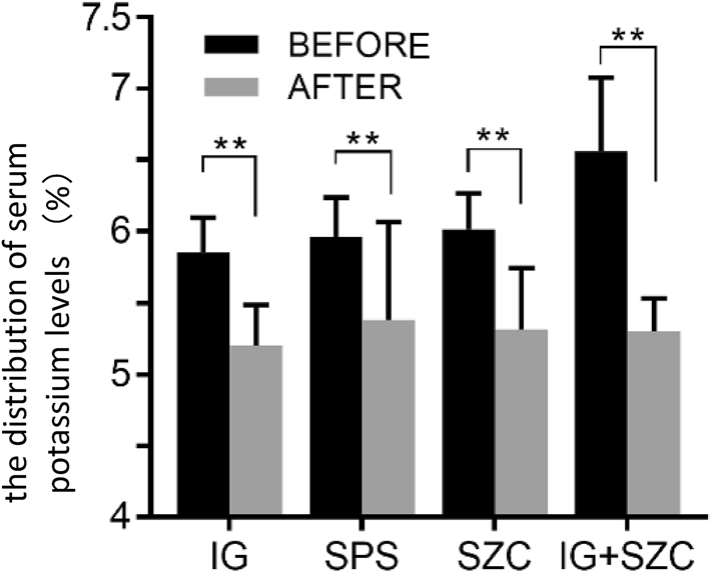
Comparison of serum potassium levels before and after treatment in each group, **P < 0.01

**Fig. 6:**
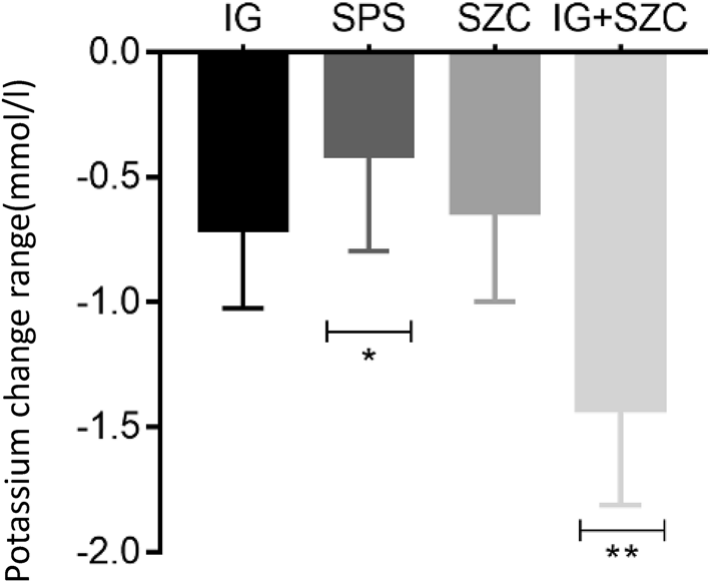
Comparison of serum potassium decrease in each group, ** means *P* < 0.01 for IG + SZC compared with the other 3 groups in the pairwise comparison, *P < 0.05 for SPS compared with the other 3 groups in the pairwise comparison

The serum potassium level of each group decreased significantly after the treatment (*P* < 0.01) (Fig. 5). Figure 6 shows the change range (*t* = 36.94, *P* < 0.01) and change rate of serum potassium (*t* = 28.25, *P* < 0.01) in each group after treatment that exhibited significant differences. The serum potassium in the IG + SZC group decreased by 1.43 ± 0.38 mmol/L (21.51 ± 5.74%), significantly higher than the remaining groups (*P* < 0.01). The decrease of serum potassium of SPS group was the lowest i.e., 0.43 ± 0.38 mmol/L (7.12 ± 6.32%), which was significantly lower than the other three groups (*P* < 0.05), and there was no statistical significance between IG group and SZC group in the changes of serum potassium level (*P* = 0.374). The SPS group had the largest CV (89.3%) compared with other groups (Table 2).

### Secondary efficacy outcome: the standard-reaching rate of serum potassium level

**Fig. 7:**
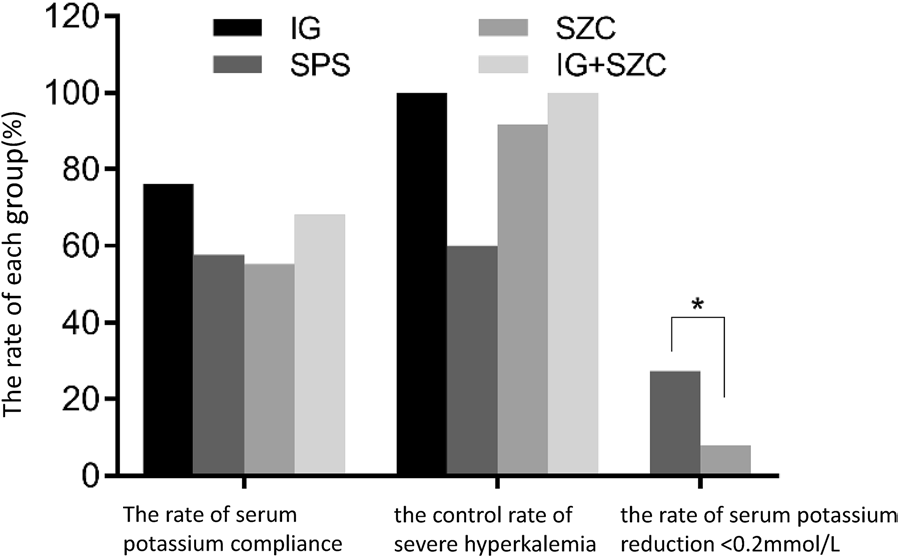
The rate of serum potassium compliance, the control rate of severe hyperkalemia and the rate of serum potassium reduction < 0.2 mmol/L in each group, *P < 0.05

After potassium lowering treatment, there was no significant difference in the rate of reaching the standard of serum potassium among groups (*t*^2^ = 4.95, *P* = 0.176) (Fig. 7). The control rate of severe hyperkalemia in SZC group was higher than that in SPS group, but the difference was not statistically significant perhaps due to the number of cases (*P* = 0.135). (Table 2).

### Associated adverse events

There was no difference in the changes of serum sodium level during potassium lowering treatment among groups (Table 3). Serum calcium levels in IG group, SPS group and IG + SZC group decreased significantly after treatment (*P* < 0.01). Serum magnesium levels of SPS group and IG + SZC group were significantly decreased after treatment (*P* < 0.01). However, there was no significant difference in the changes of serum sodium, calcium and magnesium levels in SZC group (*P* > 0.05).

**Table 3:** Changes of serum sodium, calcium and magnesium (mmol/L) before and after treatment

	IG group	SPS group	SZC group	IG + SZC group	*P*
Serum sodium before treatment	138.0 (135.0,141.0)	138.0 (136.0,139.5)	138.0 (136.0,140.0)	136.5 (135.0,139.25)	0.724
Serum sodium after treatment	139.0 (134.0,140.25)	138.0 (135.0,139.0)	137.0 (135.0, 139.25)	136.5 (134.75,140.0)	0.679
Decrease of serum sodium	0.0 (− 1.0,2.0)	0.0 (− 1.0,1.0)	0.0 (− 1.0, 3.0)	0.0 (− 2.25,3.0)	0.815
Serum calcium before treatment	2.33 (2.20,2.45)	2.20 (2.04, 2.45)	2.27 (2.04, 2.42)	2.29 (2.21,2.43)	0.291
Serum calcium after treatment	2.27^c^ (2.17,2.37)	2.22^c^ (1.98, 2.36)	2.23 (2.02, 2.38)	2.18^c^ (2.12,2.35)	0.317
Decrease of serum calcium	0.05 (0.0,0.11)	0.05 (0.03, 0.12)	0.05 (− 0.03, 0.10)	0.07 (0.02,0.15)	0.665
Serum magnesium before treatment	1.15 (1.07,1.27)	1.08 (1.15, 1.31)	1.20 (1.05, 1.36)	1.25 (1.15,1.33)	0.349
Serum magnesium after treatment	1.10 (1.05,1.29)	1.10^c^ (1.03, 1.27)	1.20 (1.09, 1.33)	1.20^c^ (1.07,1.30)	0.663
Decrease of serum magnesium	0.03 (− 0.03,0.06)	0.05 (− 0.02, 0.08)	0.03 (− 0.03, 0.08)	0.06 (− 0.02,0.09)	0.398

^a^There was no significant difference between IG and SZC groups in pairwise comparison, and the rest were statistically significant (*P* < 0.01)

^b^In pairwise comparison, there was no significant difference except IG group and SPS group (*P* < 0.05)

^c^Statistically significant difference compared with pretreatment (*P* < 0.01)

^d^*P* value of SPS group compared with SZC group

During potassium-lowering treatment, 6 cases (13.0%) of the hypoglycemia reactions occurred in the IG group, of which 4 cases (8.7%) were non-diabetic patients, 2 cases (4.3%) were diabetic patients. Also, there were 2 cases (9.0%) with symptomatic hypoglycemia in the IG + SZC group. There were no evident adverse reactions in observed in the SZC and SPS groups.

## Discussion

Hyperkalemia is a common complication in patients undergoing MHD treatment, which may result in malignant arrhythmias and sudden death [2, 15]. Moreover, acute hyperkalemia increases the risk of cardiovascular death, emergency hospitalization and the overall medical burden of MHD patients [15, 16]. The incidence of hyperkalemia is higher in patients with long interval dialysis, inadequate dialysis or interrupted dialysis is referred when the hemodialysis treatment is temporarily paused to allow the patient to attend the bathroom. Vascular access dysfunction is one of the most common reasons for discontinuing dialysis in MHD patients. Therefore, the retrospective RWS mainly included MHD patients who had to discontinue dialysis due to vascular access dysfunction.

Increasing the excretion of potassium is the fundamental measure of emergency potassium-lowing strategy, including diuretics, emergency dialysis and intestinal potassium excretion drugs. Generally, diuretics was inefficacious in excreting potassium in most MHD patients due to poor renal function and insufficient potassium excretion capacity [17]. Some MHD patients with hyperkalemia could not obtain dialysis timely due to dysfunctional access or other reasons. Therefore, the use of intestinal potassium excreting drugs or glucose plus insulin to promote the transfer of potassium ions into cells have gained much attention for the treatment of hyperkalemia in most MHD patients. When serum potassium drops to the safe range, receiving HD after repairing procedures become the common treatment measure for most MHD patients with access dysfunction. During the treatment of hyperkalemia, the commonly used drugs include IG, SPS, SZC or their combinations. However, to the best of our knowledge, there is no reported comparative study on the efficacy and safety of these regimens in MHD patients with acute hyperkalemia.

In this study, 139 MHD patients with acute hyperkalemia were enrolled, most of them with poor vascular access function. The four different kinds of potassium reduction regimens were applied i.e., IG, SPS, SZC or IG + SZC. The baseline serum potassium level and the proportion of patients with serum potassium > 6.5 mmol/L in the IG + SZC group was significantly higher than those in the other three groups with the great apprehension that severe hyperkalemia may not be corrected by a single potassium lowering regimen, and for the sake of safety, two drugs were combined to strengthen the reduction of serum potassium.

The mean decrease of serum potassium in MHD patients after 2 h of application of SZC was 0.64 ± 0.36 mmol/L, which is consistent with the previously reported studies [18], indicating that SZC can rapidly reduce serum potassium levels in MHD patients during the correction period of acute hyperkalemia. SZC is an inorganic cation exchanger whose unique structure enables it to effectively simulate physiological potassium channels in vivo, highly select serum potassium and accurately capture serum potassium. Its selectivity to serum potassium ions is about twenty-five times more to that of serum calcium and magnesium ions [18, 19]. In an environment that simulates the human gastrointestinal tract, SZC works in combination with serum potassium in the stomach and continues to function throughout the gastrointestinal tract, allowing a drop in serum potassium levels to be observed within 1 h of taking the drug [19]. After 2 h of treatment, there were no significant differences between SZC group and IG group in terms of the rate of serum potassium decline, the standard-reaching rate of serum potassium and the control rate of severe hyperkalemia. However, the IG group requires intravenous infusion, and there is a risk of hypoglycemic reaction. The IG + SZC group had the largest decrease in serum potassium of all the groups. Although the basal potassium level was the highest, the IG + SZC group had a relatively high decrease in serum potassium, the standard-reaching rate of serum potassium, and the control rate of severe hyperkalemia, indicating that SZC combined with IG application can exert a greater potassium-lowering effect. The standard-reaching rate of serum potassium in SPS group was similar to that in SZC group, but the decrease of serum potassium and the control rate of severe hyperkalemia in SPS group were significantly lower than those in SZC group. In addition, the CV of serum potassium decline in SPS group was the largest, suggesting that the efficacy of SPS in the correction period of acute hyperkalemia in MHD patients has great individual differences.

After taking SZC, there were no significant changes in serum sodium, calcium and magnesium, and no serious adverse events in MHD patients in this study, which was consistent with the results of previous clinical trials [11, 20]. However, there were 6 (13.0%) and 2 (9.0%) patients in IG group and IG + SZC groups, respectively with symptomatic hypoglycemia. In SPS group, serum calcium and magnesium decreased significantly, which was related to the non-selective cation exchange of polymer exchange resins.

There are some limitations in this study. Firstly, this study is a retrospective RWS with few selected cases. Secondly, considering the serious consequences of acute hyperkalemia and the safety of patients, all the subjects were not randomly grouped, but treatment options were chosen according to the clinical judgement. In addition, this study was of relatively short duration for it only compared the effect of 2 h after administration in MHD patients. Therefore, additional studies need to be included for rigorous, multi-center prospective intervention study in patients receiving MHD, although previous studies in the non-dialysis population have demonstrated that the efficacy and safety of SZC.

## Conclusion

To conclude, in our opinion this retrospective RWS suggested that in the correction of acute hyperkalemia among MHD patients, SZC had the similar efficacy as IG intravenous potassium-lowering, and had more advantages on convenience and safety. Combined SZC and IG increased the efficacy of potassium-lowering and the standard-reaching rate of serum potassium.

## Supplementary Information

**Additional file 1: Figure S1.** Scheme of selection of potassium-lowering regimens for enrolled patients.

## Data Availability

All data will be made available upon request.

## Abbreviations

MHD: Maintenance hemodialysis
IG: Insulin and glucose
SPS: Sodium polystyrene sulfonate
SZC: Sodium zirconium cyclosilicate
CKD: Chronic kidney disease
HD: Hemodialysis
RWS: Real-world study
ACEI: Angiotensin converting enzyme inhibitor
ARB: Angiotensin II receptor antagonist
MRA: Mineralocorticoid receptor antagonist
