# Supplementary figures and images for: Effects of different potassium-lowering regimens on acute hyperkalemia in hemodialysis patients: a real-world, retrospective study
Source: J Transl Med. 2022 Jul 25;20:333. doi: 10.1186/s12967-022-03530-4 (PMC9310460; doi:10.1186/s12967-022-03530-4)

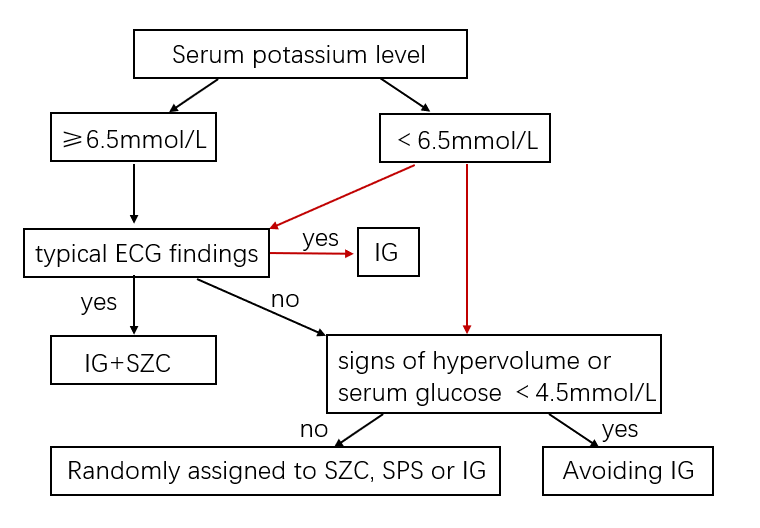


**Figure S1. Scheme of selection of potassium-lowering regimens for enrolled patients**

Supplement: Supplementary file 1 — Additional file 1: Figure S1. Scheme of selection of potassium-lowering regimens for enrolled patients. [file 12967_2022_3530_MOESM1_ESM.docx]
